# RMalign: an RNA structural alignment tool based on a novel scoring function RMscore

**DOI:** 10.1186/s12864-019-5631-3

**Published:** 2019-04-08

**Authors:** Jinfang Zheng, Juan Xie, Xu Hong, Shiyong Liu

**Affiliations:** School of Physics, Huazhong University of Science and Technology, Wuhan, 430074 Hubei China

**Keywords:** RNA structural alignment, RMalign, RMscore, Protein-RNA interaction

## Abstract

**Background:**

RNA-protein 3D complex structure prediction is still challenging. Recently, a template-based approach PRIME is proposed in our team to build RNA-protein 3D complex structure models with a higher success rate than computational docking software. However, scoring function of RNA alignment algorithm SARA in PRIME is size-dependent, which limits its ability to detect templates in some cases.

**Results:**

Herein, we developed a novel RNA 3D structural alignment approach RMalign, which is based on a size-independent scoring function RMscore. The parameter in RMscore is then optimized in randomly selected RNA pairs and phase transition points (from dissimilar to similar) are determined in another randomly selected RNA pairs. In tRNA benchmarking, the precision of RMscore is higher than that of SARAscore (0.88 and 0.78, respectively) with phase transition points. In balance-FSCOR benchmarking, RMalign performed as good as ESA-RNA with a non-normalized score measuring RNA structural similarity. In balance-x-FSCOR benchmarking, RMalign achieves much better than a state-of-the-art RNA 3D structural alignment approach SARA due to a size-independent scoring function. Take the advantage of RMalign, we update our RNA-protein modeling approach PRIME to version 2.0. The PRIME2.0 significantly improves about 10% success rate than PRIME.

**Conclusion:**

Based on a size-independent scoring function RMscore, a novel RNA 3D structural alignment approach RMalign is developed and integrated into PRIME2.0, which could be useful for the biological community in modeling protein-RNA interaction.

**Electronic supplementary material:**

The online version of this article (10.1186/s12864-019-5631-3) contains supplementary material, which is available to authorized users.

## Background

RNA plays important roles in many biological processes such as gene regulation, subcellular location and splicing. High-throughput global mapping of RNA duplexes with near base-pair resolution reveals that RNA interacts with RNA and RNA-binding proteins using higher order architectures in living cell [1]. Most of them, though their binding sites and binding regions [2] are determined, atomic interaction details are still missing, which is key to understanding molecular mechanisms underlying the RNA-RNA or RNA-protein recognition. With the increasing RNA and RNA-protein 3D structures deposited in PDB [3], it is important to develop better bioinformatics tools to compare RNA structures, which could provide a possible way to build atomic RNA-RNA or RNA-protein interaction models by inferring RNA structural homologs with lower sequence similarity. Some RNA structure comparing approaches have been developed under different scoring strategies with a traditional sequence alignment algorithm [4–7]. In these approaches, the RNA 3D structures are represented with structural alphabet (SA) [5–7] or dihedral angles [4]. Then DP algorithm is used to align RNA sequence with a substitution scoring matrix. Besides, STAR3D employs a substitution scoring function which includes RMSD, aligned stack regions and the distance [8]. SETTER is a secondary structure-based tertiary structure comparing algorithm which employs the non-overlapping generalized secondary structure unites (GSSUs) [9–11]. In the other state-of-the-art alignment approaches, SARA applies a statistical scoring function to measure the similarity of RNA 3D structures [12, 13]; and ESA-RNA uses the geodesic distance integrating RNA sequence with 3D structure information to measure the RNA similarity [14, 15]. Like using a geometric concept in ESA-RNA, R3D Align and FR3D employ geometric discrepancy to measure the RNA similarity [16, 17]. CLICK is a topology-independent tool comparing of 3D structures without a scoring function measuring the structural similarity [18, 19]. Similar to SARA-Coffee [20] coupling with sequence alignments, SupeRNAlign iteratively superimposes the RNA fragment structures with R3D and maximizes the local fit [21]. They found that R3D is scoring the best among the tools without ESA-RNA in benchmark. Based on SARA, a template-based approach PRIME is proposed in our team to build RNA-protein complex 3D structure models, which shows a higher success rate than computational docking software. However, the scoring function of RNA alignment algorithm SARA is size-dependent, which limits its ability to detect potential templates in some cases.

In this manuscript, we introduce an RNA structural alignment approach based on RMscore, which is a size independent scoring function to measure RNA structural similarity. Firstly, we reveal the liner relationship between the logarithmic length of RNA and the logarithmic radius of gyration (Rg) of RNA. At the same time, the aligned correlation coefficient (ACC) describing the relationship between RMSD and Rg also has a complex function relation with the RNA length. Combining these function relations, a length slightly independent scoring function RMscore is determined (the RMscore only slightly decreases as the length increases). Then RMscore is applied to two randomly selected independent datasets to optimize parameters and determine the transition point from similar to dissimilar. With the transition point, RMscore performs better than SARAscore [5, 13, 22] in selecting similar tRNA pairs. Then based on the RMscore, we develop an RNA structural alignment method RMalign. In RNA function classification, RMalign performs as good as ESA-RNA in balance-FSCOR. However, RNAs share the structural similarity may have different functions. So, we benchmark RMalign in structural classification. In RNA structural classification, RMalign performs much better than SARA in balance-x-FSCOR. Finally, PRIME is updated to PRIME 2.0 by replacing SARA with RMalign. PRIME 2.0 improves the success rate about 10% than previous when it is tested in protein-RNA docking benchmark.

## Methods

### Datasets

We download RNA structure coordinates from PDB [3] website with RNA structures containing at least one RNA chain. This step obtains 2557 RNA structures. Based on these RNA structures, vary datasets are constructed for variable goals. PDB-3775 is constructed to explore the relationship between the Rg of RNA and the length. Fragment-pairs dataset is built to study the relationship between the ACC and RMSD in RNA. Results in PDB-3775 and fragment-pairs are combined to estimate the expression of RMscore. To calculate all-to-all alignments of 3775 RNAs is a time-consuming process, so we randomly select two RNA-RNA pair datasets (random pairs-0.3 M and random pairs-0.1 M) without overlap. Random pairs-0.3 M and random pairs-0.1 M are built to optimize the compensation and determine the transition point, respectively. Like benchmarking modeRNA in tRNA [23], tRNA-pairs are also constructed for benchmarking RMscore. Balance-FSCOR and Balance-x-FSCOR are established to benchmark RMalign in function and structural classification. The unbound protein-RNA docking set is employed to compare the performance of PRIME 2.0 and PRIME.

### PDB-3775

Total 2557 RNA structures and their complexes from PDB are separated by chains. 3775 RNA chains are kept expect the RNA structures in mmcif format. PDB-3775 represents all RNA structures in PDB. The relationships between the Rg of RNA and the RNA length are explored in this dataset.

### Fragment-pairs

ACC in proteins describing the relationship between RMSD and Rg is reported in [24]. In order to study the relationship between the ACC and RMSD in RNA, we generate a fragment pair dataset based on PDB-3775. Only one fragment is randomly chosen for each RNA chain in PDB-3775. Then all the fragments with the identical length are made in pairs. This strategy generating structure fragments is previous used in the protein field [25].

### Random pairs-0.3 M

We randomly selected 0.3 million RNA pairs from all-to-all alignment of RNA chains in PDB-3775 to optimize parameters in RMscore. The alignment of the paired RNA is generated by needle [26]. This dataset is named as random pairs-0.3 M.

### Random pairs-0.1 M

We randomly chose 0.1 million RNA pairs from all-to-all pair of RNA chains in PDB-3775 to determine the phase transition. The alignment of the paired RNA is aligned by SARA [5, 13], which is an RNA structural alignment protocol based on unit-vector root-mean-square. This dataset is named as random pairs-0.1 M.

### tRNA-pairs

We downloaded all tRNA structures from NDB [27] (http://ndbserver.rutgers.edu/). We extract one RNA chain from one structure or its complex. This process outputs 175 RNA chains. tRNA pairs are then constructed through all-to-all pairwise alignment by SARA.

### Balance-FSCOR

FSCOR [5, 13] is downloaded from this website (http://structure.biofold.org/sara/datasets.html), which is constructed from SCOR [28] to benchmark RNA structural alignment methods. Positive pairs are generated from the RNAs with the same function in FSCOR. Negative pairs are generated from randomly selected the RNAs with different functions. The number of negative pairs is equal to the number of positive pairs. This dataset including both negative pairs and positive pairs is named as balance-FSCOR.

### Balance-x-FSCOR

Structural similarity is used as evaluation in protein structural alignment protocol. However, RNA function is used as the metric in benchmarking in balance-FSCOR. So, we construct balance-x-FSCOR to benchmark RNA structural alignment approach employing the RNA structural similarity RMScore as a metric. Firstly, FSCOR is clustered by RMalign with different RMscore cut-offs (x = 0.4, 0.45, 0.5 … 1.0) to construct the x-FSCOR. Then 1000 positive and negative pairs are randomly selected from all-to-all pairs of x-FSCOR. These datasets are named as the balance-x-FSCOR. If the number of positive or negative pairs is less than 1000, the dataset contains less pairs. The structural classes with various cut-offs of 419 RNA chains in FSCOR can be downloaded from www.rnabinding.com/RMalign/RMalign.html. The vary cut-offs are tried, because it is still unknown which value is appropriate to cluster the RNA structures.

### Unbound protein-RNA docking set

The unbound set is used to compare the performance of PRIME [22] and PRIME (2.0) in predicting protein-RNA complex structures. This set includes 49 protein-RNA structures from protein-RNA docking benchmark [29].

#### Relationship between the Rg of RNA and the RNA length

The Rg of protein is an important metric to describe the compactness of protein. Previous studies [30, 31] about protein reveal a scaling law Rg ∝ N^0.4^ where N is the number of residues in a protein. Adopting a similar strategy with protein, we investigate the relationship between Rg of the RNA and its length. Simply, all RNA structures are represented with C3’ atoms. The average log Rg located in the same length bins is calculated. After calculating Rg of all RNAs in PDB-3775, we observe that a scaling law Rg ∝ N^0.39^ for RNA.

#### Relationship between the ACC and the RNA length

The ACC has a function correlation with the Rg of protein and RMSD [24]. The Rg of the protein and RMSD also depend on the number of residues and the aligned length. Like protein, the relationship between RMSD of two aligned RNA structures of identical length and the Rg of these two can be written as Eq.1 by Zhang and Skolnick [32]:

1 $$ \mathrm{RMSD}=\sqrt{{\mathrm{R}}_{\mathrm{A}}^2+{\mathrm{R}}_{\mathrm{B}}^2-2{\mathrm{R}}_{\mathrm{A}}{\mathrm{R}}_{\mathrm{B}}\frac{\sum_{\mathrm{i}}\left({\mathbf{r}}_{\mathbf{Ai}}\bullet {\mathbf{r}}_{\mathbf{Bi}}\right)}{\sqrt{\sum_{\mathrm{i}}{{\mathbf{r}}^2}_{\mathrm{A}\mathrm{i}}{\sum}_{\mathrm{i}}{{\mathbf{r}}^2}_{\mathrm{B}\mathrm{i}}}}} $$

where R_A_ (R_B_) is the Rg for structure A (B), **r**_Ai_(**r**_Bi_) is the coordinate vector after superposition.

$$ \mathrm{ACC}=\frac{\sum_{\mathrm{i}}\left({\mathbf{r}}_{\mathrm{Ai}}\bullet {\mathbf{r}}_{\mathrm{Bi}}\right)}{\sqrt{\sum_{\mathrm{i}}{{\mathbf{r}}^2}_{\mathrm{Ai}}{\sum}_{\mathrm{i}}{{\mathbf{r}}^2}_{\mathrm{Bi}}}} $$

In order to reveal the relation between the ACC and aligned nucleic acids, we take a similar strategy used in TMalign [33]. Firstly, a fragment-pairs dataset is constructed. Secondly, the RMSD of RNA fragment pairs and the Rg of each fragment are calculated with the C3’ atom. Thirdly, the average and standard error of ACC are calculated if fragment pairs have the identical length. Eq. (2) reveals the function relation between the length of fragment and ACC after data is fitted.

2 $$ \frac{\sum_{\mathrm{i}}\left({\mathbf{r}}_{\mathrm{Ai}}\bullet {\mathbf{r}}_{\mathrm{Bi}}\right)}{\sqrt{\sum_{\mathrm{i}}{{\mathbf{r}}^2}_{\mathrm{Ai}}{\sum}_{\mathrm{i}}{{\mathbf{r}}^2}_{\mathrm{Bi}}}}\approx 0.51-0.76{\mathrm{L}}_{\mathrm{N}}{e}^{-{\mathrm{L}}_{\mathrm{N}}/2.0}+1.36{\mathrm{e}}^{-{\mathrm{L}}_{\mathrm{N}}/8.7} $$

Where L_N_ is the length of fragment in the fragment-pairs.

#### RMscore

Inspired by a protein scoring function TM-score, we introduce a size-independent scoring function RMscore describing the similarity of RNA structure. For an RNA alignment, RMscore is defined as:

3 $$ \mathrm{RMscore}=\operatorname{Max}\left[\frac{1}{\begin{array}{l}{\mathrm{L}}_{\mathrm{N}}\\ {}\end{array}}\sum \limits_{\mathrm{i}=1}^{{\mathrm{L}}_{\mathrm{T}}}\frac{1}{1+{\left(\frac{{\mathrm{d}}_{\mathrm{i}}}{{\mathrm{d}}_0}\right)}^2}\right] $$

Where L_N_ is the length of average of target and query RNA, L_T_ is the length of aligned nucleic acids to the target structure, d_i_ is the distance between the ith pair of aligned nucleic acids and d_0_ is a scale to normalize the length effect. ‘Max’ denotes the maximum after optimal superposition. For different scoring strategies, a different d_0_ is adopted. In RMscore, a length dependent d_0_ is adopted. Similarity to TM-score, d_0_ is estimated by multiplying $\sqrt[]{1\hbox{-} \mathrm{ACC}}$ and Rg. The relation between d_0_ and the length can be estimated from Rg ∝ N^0.39^ and eq. (2).

4 $$ {\mathrm{d}}_0\sim {{\mathrm{L}}_{\mathrm{N}}}^{0.39}\ast \sqrt{1-0.51+0.76{\mathrm{L}}_{\mathrm{N}}{\mathrm{e}}^{-{\mathrm{L}}_{\mathrm{N}}/2.0}-1.36{\mathrm{e}}^{-{\mathrm{L}}_{\mathrm{N}}/8.7}}-\mathrm{compensation} $$

Here the constant compensation (set as 0.6) is introduced to smooth the curve when the RMscore is optimized in random pairs-0.3 M (Additional file 1: Figure S1). Eq. 4 can be well approximated by a simple formula (The minimum d_0_ is optimized to smooth the curve).

5 $$ {\mathrm{d}}_0\approx \sqrt[3]{{\mathrm{L}}_{\mathrm{N}}-8.11}-0.44\left(\min {\mathrm{d}}_0=0.25\right) $$

#### Searching engine of RMscore

To find the spatially optimal superposition of the query and the target structure with the maximum RMscore according to eq. (3) and eq. (5), we use an iterative searching algorithm from TM-score.

#### Benchmark of RMscore on tRNA pairs

The tRNAs are selected to benchmark RMscore as the RNA homology modelling method modeRNA was also benchmarked in tRNA dataset [23]. For comparison of the ability to select the similar RNA structures for RMscore and SARAscore (normalized SARAscore), we determine the phase transition point of RMscore and SARAscore in random pairs-0.1 M. All alignments of RNA pairs are generated by SARA. The target SARAscore is normalized by dividing SARAscore of aligning itself. After the phase transition point from dissimilar to similar pairs is determined, RMscore and SARAscore are tested in tRNA pairs to distinguish similar (RMSD <= 5 Å) or dissimilar (RMSD > 5 Å) tRNA pairs. The alignments of tRNA pairs are also generated by SARA. A possible application of RMscore is to measure similarity between the native RNA structures and RNA models [23].

#### RMalign

**Fig. 1:**
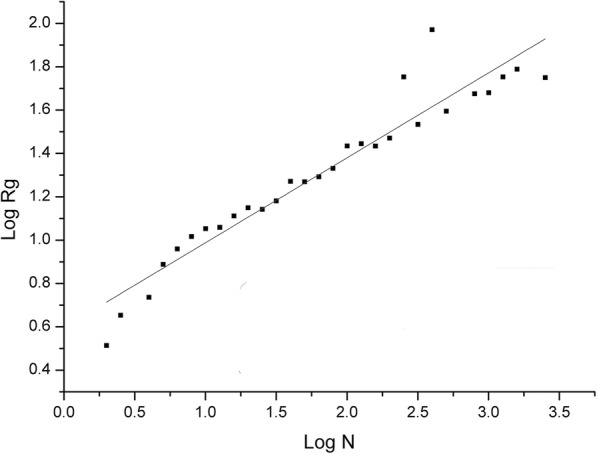
*Log Rg* vs *Log N.* The log of average Rg of RNA is plotted against log length of the corresponding RNA for 3775 RNAs. Rg is calculated with C3’ atom. The log length is split into 29 bins across the min to the max log RNA length. The Rg located in the same length bin is represented with the average value. The standard errors are not shown for that it is closed to 0. The data is fitted with a linear function (y(x) = a + b*x). Parameter of a = 0.60 ± 0.04 and b = 0.39 ± 0.02

We develop RMalign, an RNA structural alignment tool based on RMscore. A similar strategy from TM-align is taken by RMalign (Fig. 1). The process of RMalign to compare two RNAs can be divided into 4 steps.

##### Step 1:Initial structural alignment

In this step, a total of three types of alignments are used to obtain an initial alignment. They are RNA secondary structural alignment (SSA), gapless structural alignment (GSA) and alignment combining SSA and GSA, respectively.

1.1 RNA SSA. We totally consider five secondary structural states of RNA calculated by X3DNA [34]. They are stem, bulge, internal loop, hairpin loop and other, respectively. So the RNA sequence can be represented by a string consisting of 5 characters. And then DP algorithm [35] is implemented to align RNAs. The aligned nt with identical/different secondary structural state is assign to 1/0. Penalty of gap-open is set to − 1.

1.2 RNA GSA. The secondary initial structural alignment is GSA. In TMalign, the TM-score is used as the comparison metric. In RMalign, we employ the RMscore to select the best alignment.

1.3 Alignment combining SSA and GSA. In the third initial alignment, we combine the SSA and GSA with the scoring matrix that is a half/half combination of secondary score matrix (the first initial alignment) and distance score matrix (the secondary initial alignment).

##### Step 2: Scoring for an alignment

We obtain the alignments based on the step 1. In this step, the alignment is scored by the RMscore. First, the alignment is divided into fragment of length (4,8 …. LN) where LN is the length of alignment. The complete alignment fragment is then rotated by the convergent rotation matrix which is obtained by continuously superposing the nts of fragment by Kabsch algorithm with distance less than 5 Å. Secondary, the RMscore is calculated with eq.3. Then we obtained a new fragment by shifting one nt from 5′ to 3′ end and the rotation process is repeated until the fragment reaches the end of 3′. All the possible fragments are tried. Finally, the rotation matrix with the best RMscore is kept.

##### Step 3: Update the alignment

We obtain the RMscore rotation matrix from step 2. In this step, the aligned RNA structure is rotated by the RMscore rotation matrix. And then a scoring similarity matrix S(i, j) is calculated according to the Eq.6. The new alignment is obtained by DP algorithm [35] with the scoring similarity matrix and a gap-opening penalty of − 0.6. If the new alignment is equal to the previous alignment, then the process returns to step 2. Otherwise the process goes to step 4.

##### Step 4: Final scoring and output

In this step, the alignment is scored with all the aligned nts. And the result is output.

Comparing with TMalign, two processes are modified. Firstly, in the secondary type of initial alignment, RNA secondary structure is calculated by X3DNA [34]. Secondly, all the aligned nucleic acids are used to score instead of setting a distance cut-off in the final scoring process.

6 $$ S\left(i,j\right)=\frac{1}{1+{d_{ij}}^2/{d}_0{\left({L}_{\mathrm{min}}\right)}^2} $$

where d _ij_ is the distance of the ith nt in RNA 1 and the jth nt in RNA 2 under the RMscore superposition. The value of d_0_ is determined by eq. 5. The L_min_ is the length of smaller RNA.

#### Benchmark of RMalign on balance-FSCOR and balance-x-FSCOR

To test the performance of RMalign in RNA function classification and compare with ESA-RNA, a balance-FSCOR based on FSCOR is constructed. The same structures may have different functions. And the purpose of RNA structural alignment approach is to detect the structural similarity. So, we also benchmark RMalign in balance-x-FSCOR. The AUC value is used as the metric to measure the performance.

#### Predicting protein-RNA 3D structure

We previous developed an approach PRIME [22] to predict the protein-RNA 3D structure. PRIME is tested on an unbound protein-RNA docking benchmark. The result shows that PRIME performs better than 3dRPC [36]. We update previous PRIME to v2.0, because RMalign performs better than SARA in balance-x-FSCOR. A similar approach in PRIME is adopted to build the protein-RNA complex structure model. The transformation matrices of TM-score and RMscore are applied to superimpose the target protein and RNA onto the templates. The ligand RMSD of RNA C3’ atom between the model and the native structure is calculated. The quality of the model is measured by ligand RMSD. A prediction defined as “acceptable” for the ligand RMSD <= 10 Å [37].

## Results

### Principle and benchmark of RMscore

**Fig. 2:**
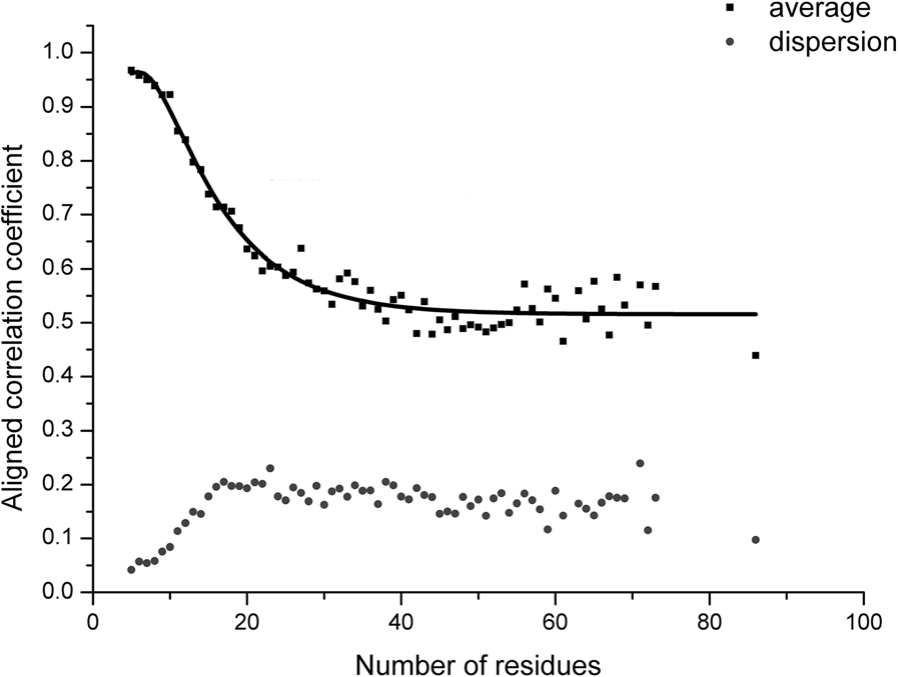
*Aligned correlation coefficient* vs *number of residues*. Average aligned correlation coefficient is plotted against number of residues. The data is calculated from RNA fragment pairs. Because the distribution of length of RNA is dispersive, we don’t count length bins containing fewer than 20 RNA fragment pairs. The square points are the average of aligned correlation coefficient. These points are fitted with nonlinear function (y(x) = E – A*x*exp.(x/B) + C*exp.(x/D)). Parameters of A = 0.76 ± 0.29, B = − 2.02 ± 0.57, C = 1.36 ± 0.41, D = − 8.73 ± 1.30 and E = 0.52 ± 0.01. The circle points are the standard error of aligned correlation coefficient

**Fig. 3:**
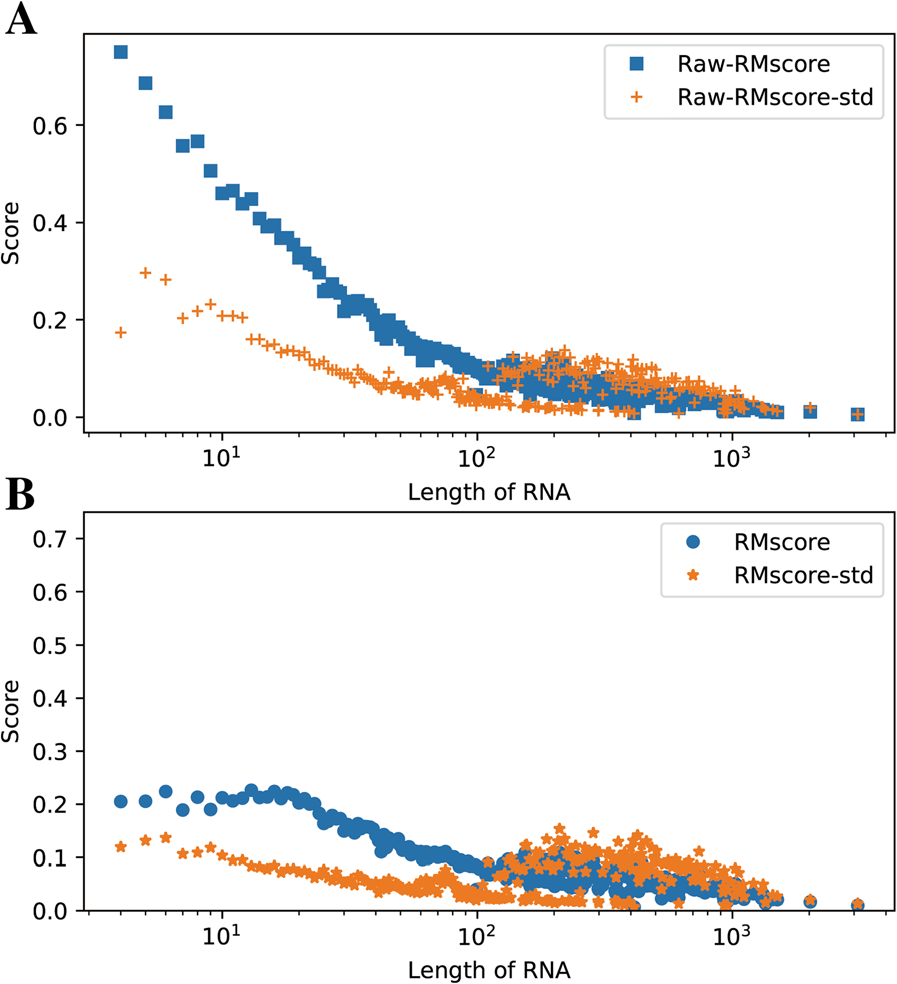
*RMscore(B)/Raw-RMscore(A)* vs *Length of RNA* . The data is calculated from 0.3 million random selected pairs. RNA sequence alignment is accomplished by needle in EMBOSS package. The definition of RMscore is derived from TM-score. Raw-RMscore is calculated with the definition of RMscore but d_0_ = 5. The legends with a suffix “-std” are the standard error of corresponding score

**Fig. 4:**
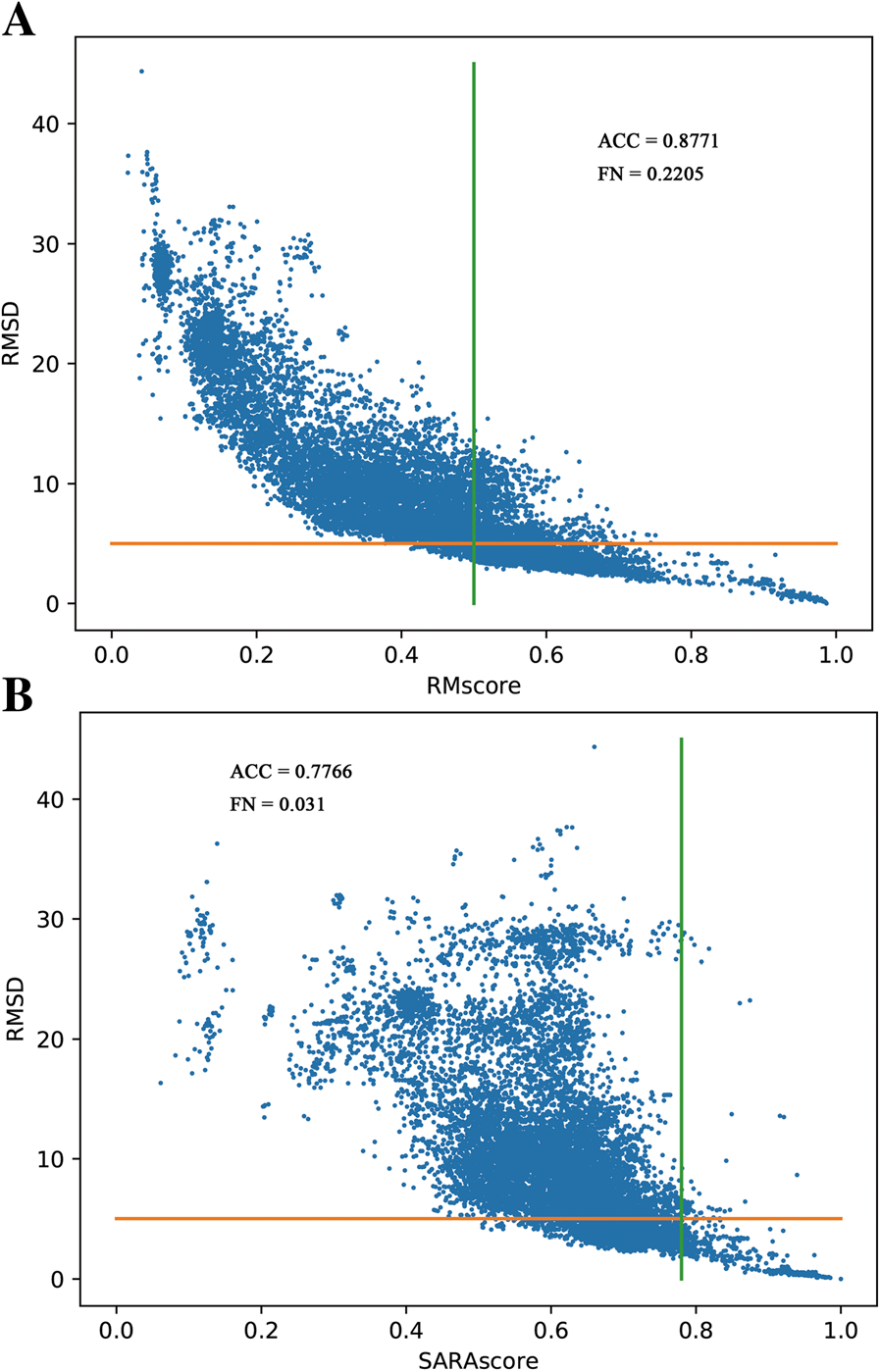
*Performance of RMscore and SARAscore in identifying similar tRNA pairs*. RMSD are plotted against RMscore (**a**) and SARAscore (**b**) for all-to-all pairwise comparison of 175 tRNA structures. Horizontal line (RMSD = 5 Å) and vertical line (score = phase transition value at 0.5 (**a**) and 0.78 (**b**)) divide the figure into 4 quadrants. Precision is defined as that the total of the number of points in upper left and the number of points in bottom right divides by total number of points. The precision of RMscore (**a**) is 0.8771 and SARAscore (**b**) is 0.7766. TPR is defined as that the number of points in bottom right divides by total number of points. The TPR of RMscore is 0.2205(**a**) and SARAscore is 0.031(**b**)

**Fig. 5:**
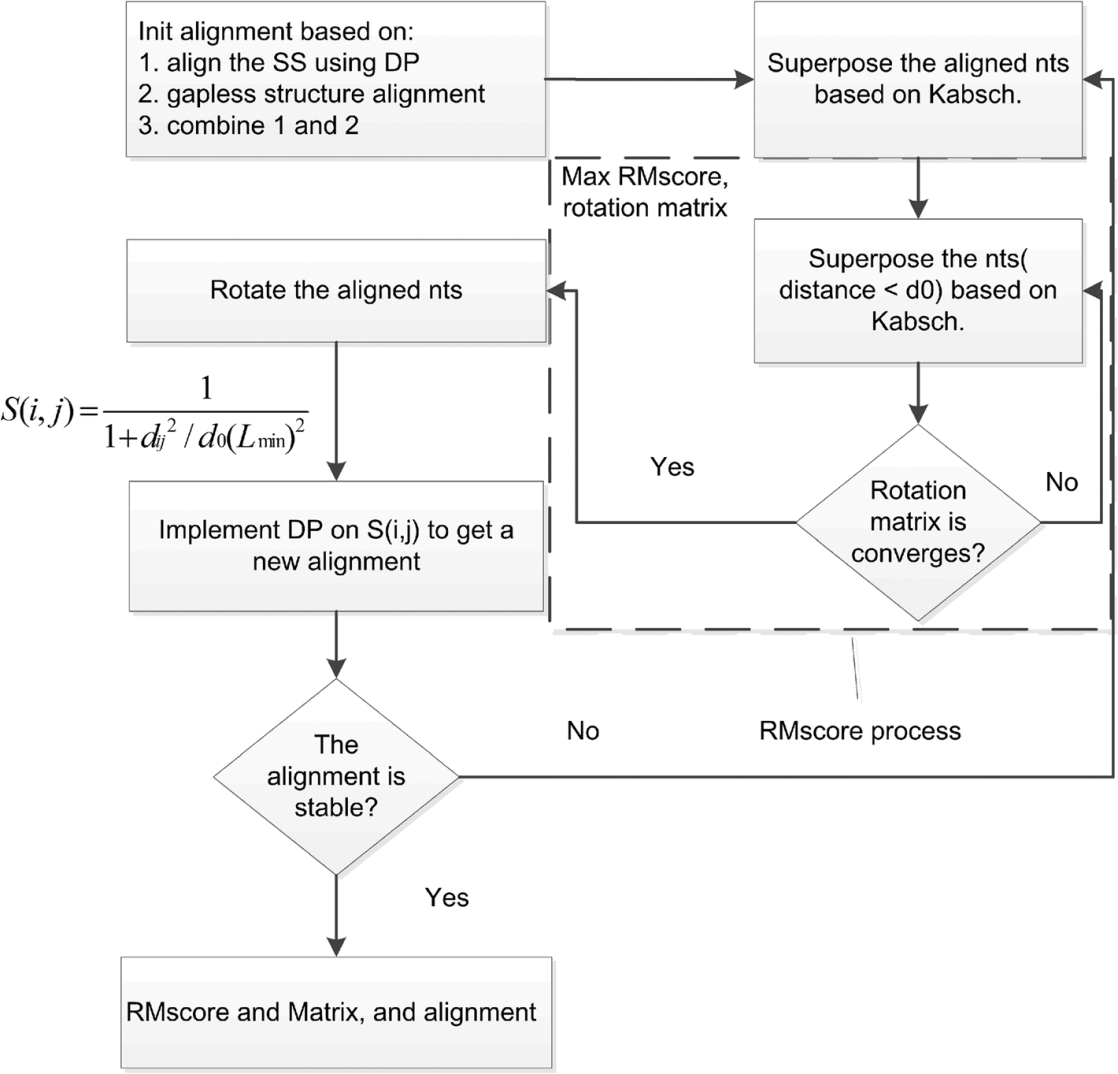
*Process of RMalign*. SS stands for secondary structure and DP stands for dynamic programming algorithm. This process is a modification of TMalign

In Fig. 2(a), it shows that the raw-RMscore (d_0_ is assigned to 5 in the definition of RMscore) changes with the length. To overcome the shortcoming of length-dependent scoring function for comparing RNA structures, we propose a size-independent scoring function RMscore (all RMscores discussed in this manuscript are normalized by an average length) to measure the RNA structural similarity. In order to obtain the formula of RMscore like TM-score, firstly, we reveal that log Rg of RNA has liner relation (R^2^ = 0.91) with the logarithmic RNA length (Fig. 3). Secondly, we found that aligned correlation coefficient has a complex function relation (R^2^ = 0.95) with the number of aligned nucleic acids (Fig. 4). Then a compensation value 0.6 is introduced to smooth the average RMscore in random pairs-0.3 M. (Additional file 1: Figure S1). The final average RMscore shows a slightly dependent on the RNA length with the highest standard error 0.2 (Fig. 2b). For comparing RMscore and normalized SARAscore, the relationship between the RMSD and the RMscore/normalized SARAscore in 0.1 million RNA pairs randomly selected from total pairs are investigated. In Additional file 2: Figure S2, it shows that the phase transition (from noise to similar RNA pairs) are 0.50 and 0.78 (at accumulative fraction = 0.5) for RMscore and normalized SARAscore, respectively. For ability of selecting similar RNA pairs with phase transition with the cut-off, RMscore and SARAscore discriminate 0.88 (Fig. 5a) and 0.78 (Fig. 5b) pairs in all-to-all pairwise structure comparison for 172 tRNA structures, respectively. The result shows the RMscore can distinguish similar (RMSD <= 5 Å) or dissimilar (RMSD > 5 Å) RNA pairs (Additional file 3: Figure S3). The above results indicate RMscore is an appropriate metric to measure RNA structural similarity.

### Benchmark of RMalign and comparison with other approaches

**Fig. 6:**
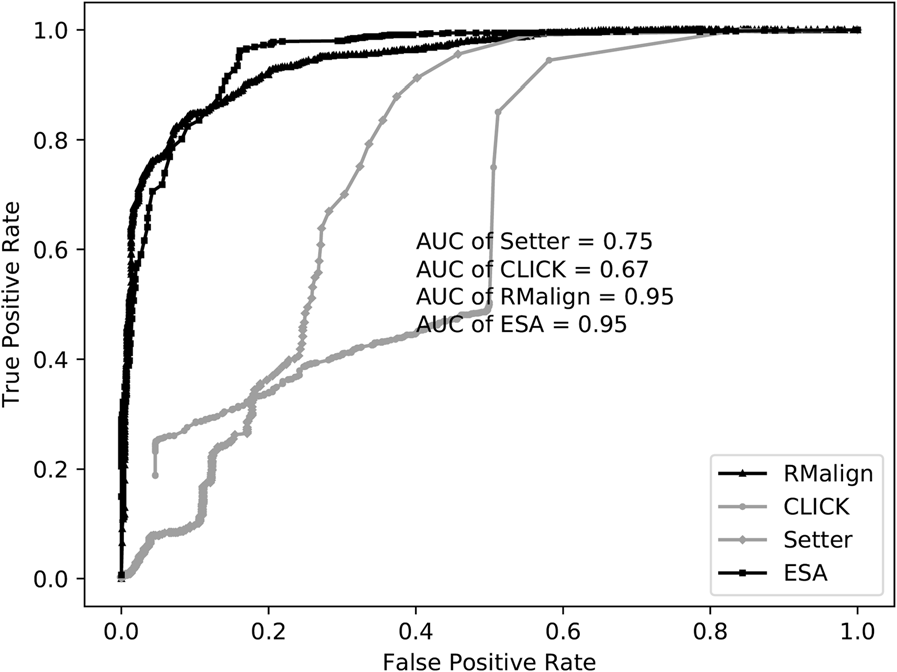
*ROC curves for benchmarking in balance-FSCOR*. The RNA pairs in the same functional class are regarded as the positive and pairs in the different functional class are regarded as the negative. The AUC of RMalign is 0.95 which is equal to ESA-RNA. The AUC of Click is 0.67. The AUC of SETTER is 0.75

For benchmarking RMalign in RNA function classification, FSCOR [13]is downloaded and then balance-FSCOR is constructed. In the research of ESA-RNA [14], many RNA structural alignment approaches have been compared in FSCOR. The result shows that ESA-RNA is the best RNA structural alignment tool. In addition, SETTER [9] employs “Distance” to measure the similarity of RNA and Click [18] does not have a scoring function. And here we present the results of comparison with ESA-RNA, SARA, SETTER and Click. RMalign obtains the AUC value of 0.95 which is as good as ESA-RNA in balance-FSCOR (Fig. 6). However, RMalign has two advantages comparing to ESA-RNA. Firstly, RMalign is written with C++ and ESA-RNA is written with a commercial software Matlab. Secondly, the geodesic distance describing the RNA structural similarity in ESA-RNA is not normalized and RMscore is a size independent score. In Additional file 4: Figure S4, it shows that the distribution of RMscore of negative and positive pairs in balance-FSCOR. This figure indicates that RMalign can distinguish negative and positive pairs clearly. In Additional file 5: Figure S5, it also shows ACC (highest value 0.88), MCC (highest value 0.73), F-measure (highest value 0.87) values of RMscore with different cut-offs. A false positive alignment example (total 13 cases with cut-off = 0.6) shows RMalign detects the RNA structural similarity but they have the different functions (Additional file 6: Figure S6). These 13 cases indicate that RNA function is not an appropriate metric to evaluate RNA structural alignment approach. For comparing the performance of RNA alignment tool with RNA structural similarity, we perform all-to-all alignment of FSCOR to re-cluster the 419 RNA chains with different RMscore x as cut-off. Then balance-x-FSCOR is constructed for comparing of RMalign and SARA in structural classification. In Additional file 7: Figure S7, it shows that AUC of RMalign is higher than AUC of SARA in balance-x-FSCOR. The performances on balance-FSCOR and balance-x-FSCOR show that RMalign could be used to predict RNA functions based on RNA structural similarity.

### Predicting protein-RNA 3D structure with PRIME 2.0 and comparison with PRIME

**Fig. 7:**
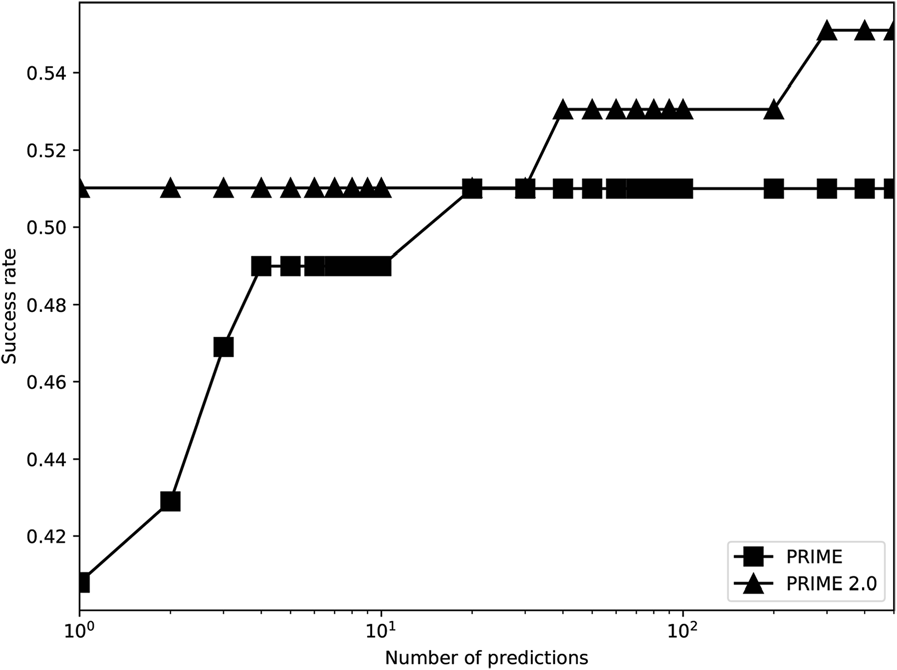
*Comparison of previous PRIME*. The template docking is performed by previous PRIME (previous PRIME) and updated PRIME (PRIME 2.0). The successful prediction is defined as at least one match with ligand RMSD <= 10 Å in top 10 and top 500 (for template docking the max prediction number is 439)

**Fig. 8:**
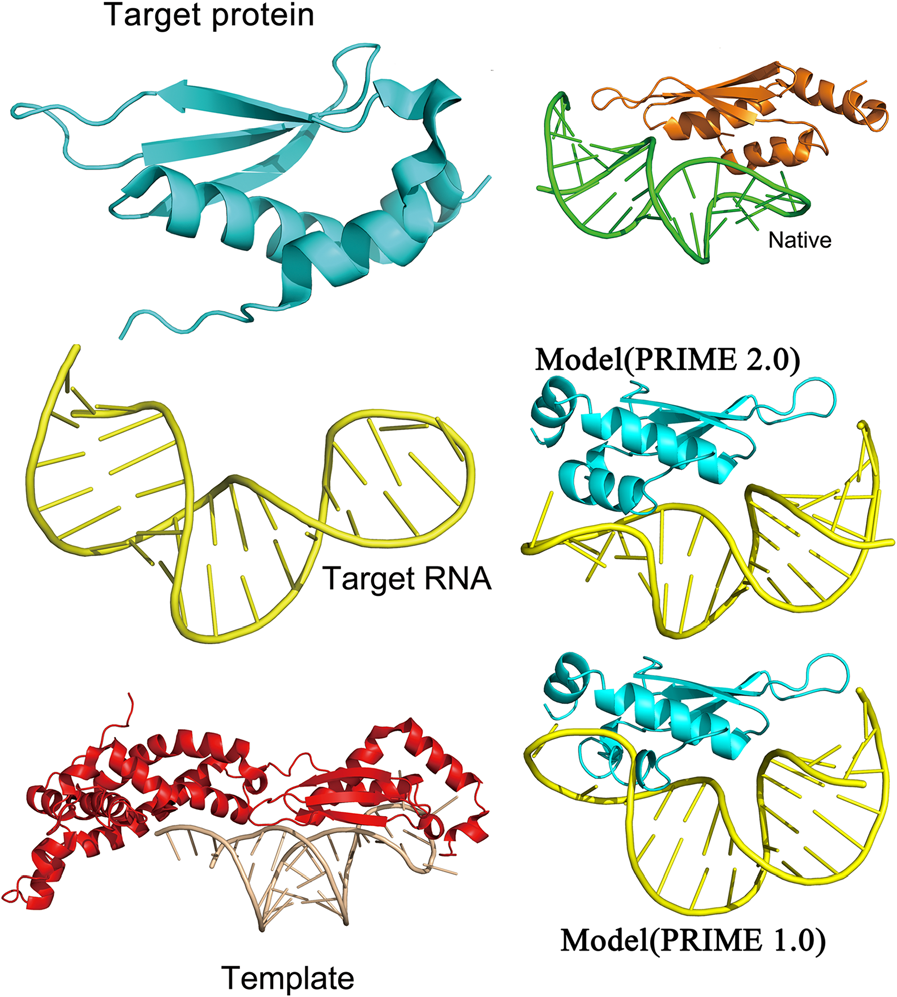
*An example of target modeled by PRIME 2.0 but PRIME fails in this case*. The target, 1t4o chain A and 1t4l chain A, is modeled on the template 4oog, chain C and D. The target/template structural similarity for protein is 0.462 (TM-score) and RNA is 0.600 (RMscore). The ligand RMSD for the model (PRIME 2.0) is 6.81 Å and for the model (PRIME 1.0) is 12.57 Å. The case shows that more templates can be detected by RMalign than SARA

For testing the ability of RMalign in detection of the protein-RNA templates, we update PRIME to PRIME 2.0 by replacing SARA with RMalign. PRIME 2.0 is tested on the unbound RNA-protein docking benchmark containing 49 complexes. In Fig. 7, it shows the docking results. For top 1 prediction, the success rate of PRIME 2.0 is about 10% higher than that of PRIME. The result indicates that RMscore can select more potential templates than SARAScore in protein-RNA 3D complex structure prediction. For the top 300 predictions, success rate of PRIME 2.0 is higher than PRIME. In Fig. 8, it shows a successful example in PRIME 2.0 but it fails in PRIME. Above results indicate than RMalign can detect more templates for protein-RNA complex structure modeling.

## Discussion

In discussion, we introduce an RNA structural alignment approach RMalign, which includes RMscore as the similarity score. The definition of RMscore is derived from TM-score which has been applied in protein structural alignment successfully. However, the RMscore shows a slightly dependent on RNA length. This phenomenon may be caused by the flexible structure of RNA. It is hard to benchmark RMscore like TM-score because that study in RNA falls behind in protein. For an example, the best way to benchmark RMscore is to compare the similarity between RNA model and native structure in RNA structure modelling. However, no related studies about size-independent scoring function have been investigated. Even more, the RNA homology modelling modeRNA employs RMSD or LG-score which is introduced as an auxiliary metric to measure the RNA structural similarity without any modifications [23]. Considering the current situation, we study the relationship between RMscore and RMSD in RNA. The result shows that RMscore = 0.5 can discriminate the similar and dissimilar structure. Benchmarking in tRNA pairs, RMscore increases with the RMSD decreasing like the relationship between protein identity and its structural similarity.

## Conclusion

In this study, we develop a novel RNA 3D structure alignment approach RMalign, which is based on a size-independent scoring function RMscore. we systematically analyzed RNA sequence and structure relationship to the binding mode, and exhaustively benchmarked the predictive modeling. The results show that in pairwise structure comparison for 172 tRNA structures RMalign significantly outperforms SARA. Replacing SARA with RMalign, the success rate of PRIME (v2.0) is 10% improved than before. Benchmarking on RNA function prediction, RMalign also shows a very high precision with AUC 0.95, which is as good as ESA. The study provides a foundation for novel RNA structural alignment approach in a size-independent way, applicable to the protein-RNA complex structure modeling and RNA function and fold classification. On the basis of the results we designed and implemented an RNA alignment tool, which should be useful for the biological community interested in RNA structural studies.

## Additional files

Additional file 1: **Figure S1.** *RNA structural similarity* vs *the length of RNA*. The length of RNA is plotted against RMscore with a different compensation (a is 0.1, b is 0.3, d is 0.6 and d is 0.9) to smooth the average RMscore at a smaller length, in random pairs 0.3 M. RNA alignment is accomplished by needle in EMBOSS package. Raw-RMscore means that d_0_ is assigned to 5 Å in RMscore definition. The compensation is chosen as 0.6 that will result in the smoothest curver. (PDF 227 kb)

Additional file 2: **Figure S2.** *RMSD* vs *RNA structural similarity*. SARAscore (A) and RMscore are plotted against RMSD in 0.1 million randomly selected pairs, RNA structural alignments are accomplished by SARA. The insets show the fraction of RNA-RNA pairs with RMSD <= 5 Å are plotted with 0.05 bins to show the phase transition from dissimilar RNA pairs to the similar pairs. (PDF 413 kb)

Additional file 3: **Figure S3.** *Distribution of RMScore (B) and SARAscore (A)*. All-to-all pairs are categorized into Low-RMScore (5 Å < RMSD <= 10 Å), High-RMscore (RMSD <= 5 Å) or Non-RMscore (RMSD > 10 Å). The same category criterion is applied for SARAscore. The RMscores corresponding to the peak value are separated clear, which can be used to distinguish similarity or dissimilarity RNA pairs, but SARAscores corresponding to the peak value are close. (PDF 178 kb)

Additional file 4: **Figure S4.** *Distribution of RMscore benchmarking in balance-FSCOR*. Positive pairs are RNA pairs with the same functions. Negative pairs are RNA pairs with different functions. The figure shows that most negative pairs have lower RMscore and most positive pairs have higher RMscore. (PDF 196 kb)

Additional file 5: **Figure S5.** *F-measure, ACC, MCC* vs *RMscore cut off*. F-measure, ACC (accuracy) and MCC are plotted against RMscore cut off selected to predict the positive or negative pairs in benchmarking on balance-FSCOR. (PDF 359 kb)

Additional file 6: **Figure S6.** *A false positive example of RMalign benchmarking in balance-SCOR*. 5msf:S (yellow) is superposed on 2iz8:R (cyan) by RMalign. These two RNA structures are very similar (RMscore = 0.837). But they are belonged to two distinct function classes in FSCOR (Phage_coat_protein_binding, MS2_phage_coat_protein_binding_stem-loop). (PDF 236 kb)

Additional file 7: **Figure S7.** *Benchmarking of RMalign and SARA in balance-x-FSCOR*. (PDF 143 kb)

## Abbreviations

AUC: Area Under Curve
GSA: Gapless structural alignment
nt: Nucleotide
SSA: RNA secondary structural alignment
