# Supplementary material for: RMalign: an RNA structural alignment tool based on a novel scoring function RMscore
Source: BMC Genomics. 2019 Apr 8;20:276. doi: 10.1186/s12864-019-5631-3 (PMC6454663; doi:10.1186/s12864-019-5631-3)
Supplement: Supplementary file 1 — Figure S1. RNA structural similarity vs the length of RNA. The length of RNA is plotted against RMscore with a different compensation (a is 0.1, b is 0.3, d is 0.6 and d is 0.9) to smooth the average RMscore at a smaller length, in random pairs 0.3 M. RNA alignment is accomplished by needle in EMBOSS package. Raw-RMscore means that d0 is assigned to 5 Å in RMscore definition. The compensation is chosen as 0.6 that will result in the smoothest curver. (PDF 227 kb) [file 12864_2019_5631_MOESM1_ESM.pdf]

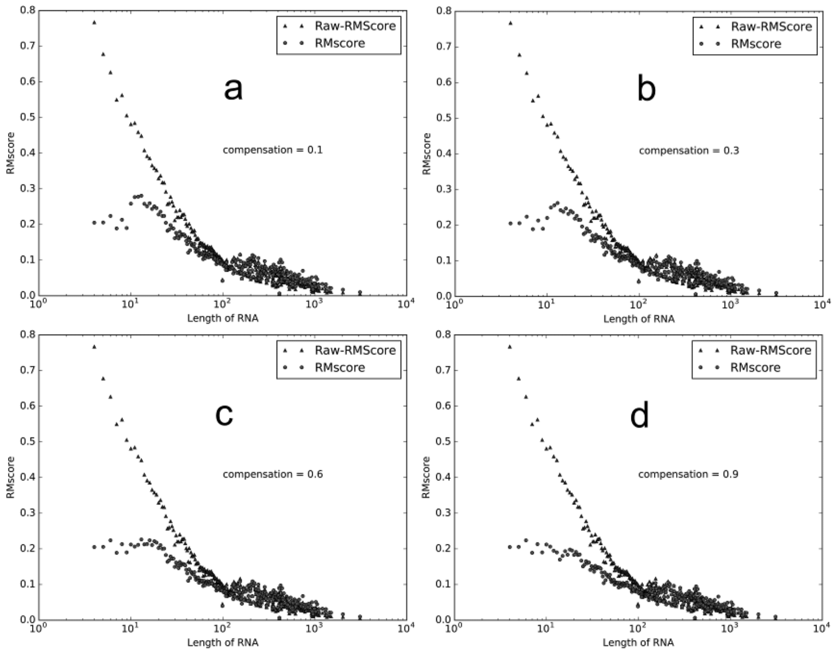

Figure S1. *RNA structural similarity vs the length of RNA.* The length of RNA is plotted against RMscore with a different compensation (a is 0.1, b is 0.3, d is 0.6 and d is 0.9) to smooth the average RMscore at a smaller length, in random pairs 0.3M. RNA alignment is accomplished by needle in EMBOSS package. Raw-RMscore means that d0 is assigned to 5 angstrom in RMscore definition. The compensation is chosen as 0.6 that will result in the smoothest curver.
