# Supplementary material for: RMalign: an RNA structural alignment tool based on a novel scoring function RMscore
Source: BMC Genomics. 2019 Apr 8;20:276. doi: 10.1186/s12864-019-5631-3 (PMC6454663; doi:10.1186/s12864-019-5631-3)
Supplement: Supplementary file 2 — Figure S2. RMSD vs RNA structural similarity. SARAscore (A) and RMscore are plotted against RMSD in 0.1 million randomly selected pairs, RNA structural alignments are accomplished by SARA. The insets show the fraction of RNA-RNA pairs with RMSD <= 5 Å are plotted with 0.05 bins to show the phase transition from dissimilar RNA pairs to the similar pairs. (PDF 413 kb) [file 12864_2019_5631_MOESM2_ESM.pdf]

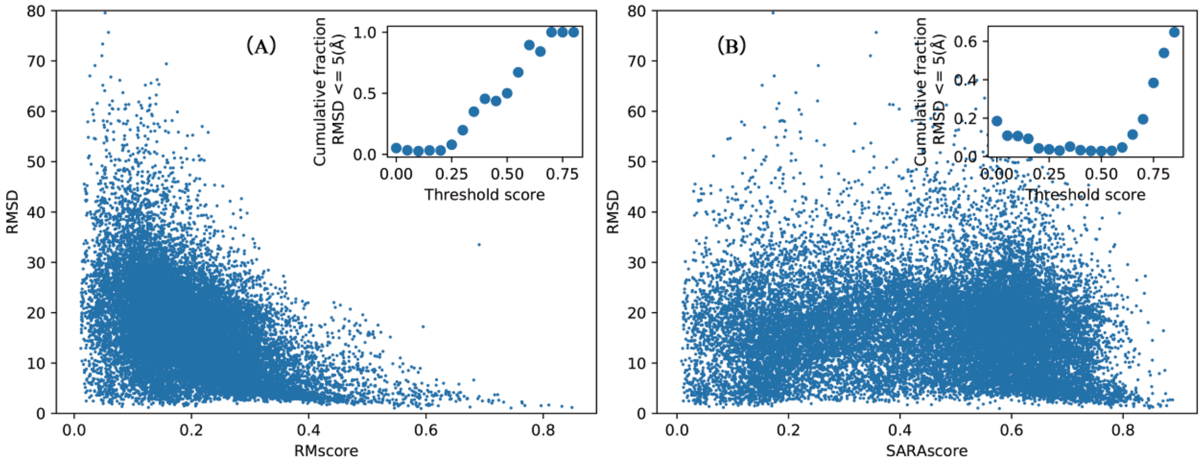


**Figure S2**. *RMSD vs RNA structural similarity*. SARAscore (A) and RMscore are plotted against RMSD in 0.1 million randomly selected pairs, RNA structural alignment are accomplished by SARA. The insets show the fraction of RNA-RNA pairs with RMSD <= 5 Å are plotted with 0.05 bins to show the phase transition from dissimilar RNA pairs to the similar pairs.
