# Supplementary material for: RMalign: an RNA structural alignment tool based on a novel scoring function RMscore
Source: BMC Genomics. 2019 Apr 8;20:276. doi: 10.1186/s12864-019-5631-3 (PMC6454663; doi:10.1186/s12864-019-5631-3)
Supplement: Supplementary file 3 — Figure S3. Distribution of RMScore (B) and SARAscore (A). All-to-all pairs are categorized into Low-RMScore (5 Å < RMSD <= 10 Å), High-RMscore (RMSD <= 5 Å) or Non-RMscore (RMSD > 10 Å). The same category criterion is applied for SARAscore. The RMscores corresponding to the peak value are separated clear, which can be used to distinguish similarity or dissimilarity RNA pairs, but SARAscores corresponding to the peak value are close. (PDF 178 kb) [file 12864_2019_5631_MOESM3_ESM.pdf]

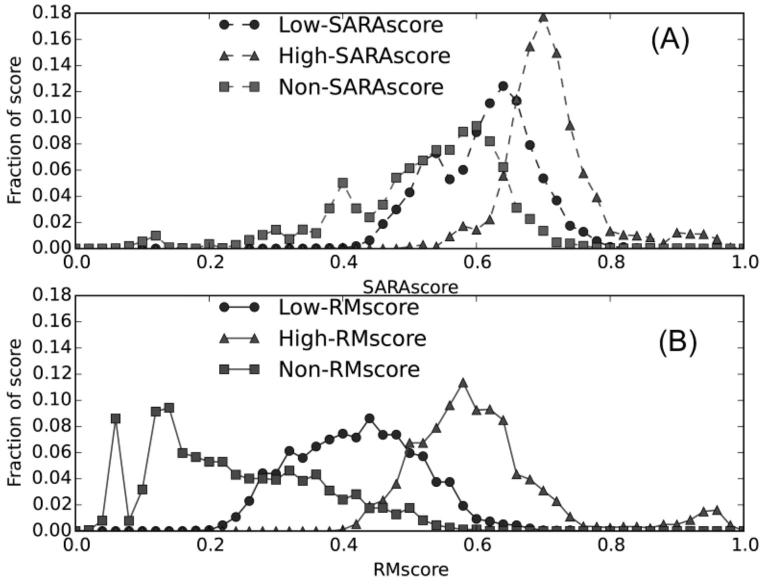

Figure S3. *Distribution of RMScore (B) and SARAscore (A)*. All-to-all pairs are categorized into Low-RMScore (5Å < RMSD <= 10Å), High-RMscore (RMSD <= 5Å) or Non-RMscore (RMSD >10Å). The same category criterion is applied for SARAscore. The RMscores corresponding to the peak value are separated clear, which can be used to distinguish similarity or dissimilarity RNA pairs, but SARAscores corresponding to the peak value are close.
