# Supplementary material for: RMalign: an RNA structural alignment tool based on a novel scoring function RMscore
Source: BMC Genomics. 2019 Apr 8;20:276. doi: 10.1186/s12864-019-5631-3 (PMC6454663; doi:10.1186/s12864-019-5631-3)
Supplement: Supplementary file 4 — Figure S4. Distribution of RMscore benchmarking in balance-FSCOR. Positive pairs are RNA pairs with the same functions. Negative pairs are RNA pairs with different functions. The figure shows that most negative pairs have lower RMscore and most positive pairs have higher RMscore. (PDF 196 kb) [file 12864_2019_5631_MOESM4_ESM.pdf]

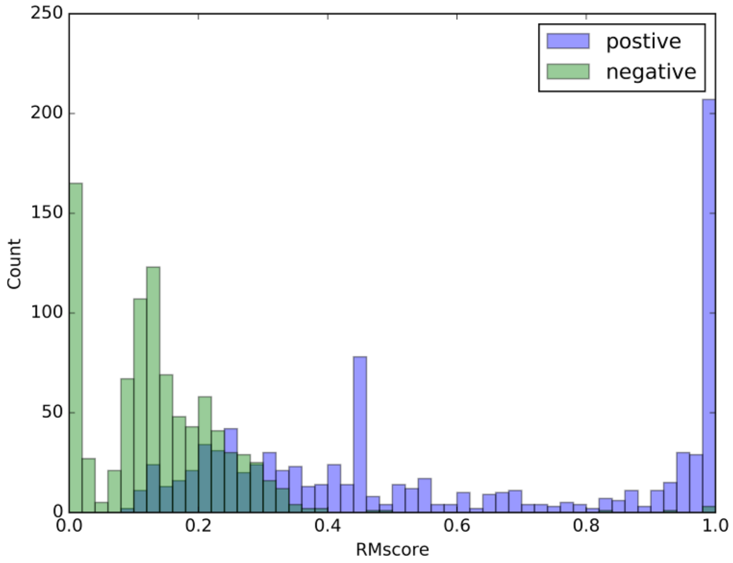

Figure S4. *Distribution of RMscore benchmarking in balance-FSCOR*. Positive pairs are RNA pairs with the same functions. Negative pairs are RNA pairs with different functions. The figure shows that most negative pairs have lower RMscore and most positive pairs have higher RMscore.
