# Supplementary material for: RMalign: an RNA structural alignment tool based on a novel scoring function RMscore
Source: BMC Genomics. 2019 Apr 8;20:276. doi: 10.1186/s12864-019-5631-3 (PMC6454663; doi:10.1186/s12864-019-5631-3)
Supplement: Supplementary file 5 — Figure S5. F-measure, ACC, MCC vs RMscore cut off. F-measure, ACC (accuracy) and MCC are plotted against RMscore cut off selected to predict the positive or negative pairs in benchmarking on balance-FSCOR. (PDF 359 kb) [file 12864_2019_5631_MOESM5_ESM.pdf]

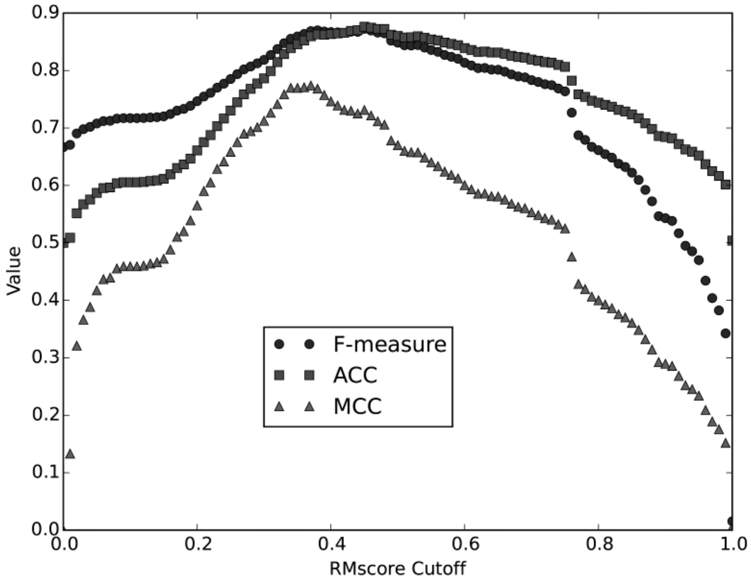

Figure S5. *F-measure, ACC, MCC vs RMscore cut-off*. F-measure, ACC (accuracy) and MCC are plotted against RMscore cut-off selected to predict the positive or negative pairs in benchmarking on balance-FSCOR.
