# Supplementary material for: RMalign: an RNA structural alignment tool based on a novel scoring function RMscore
Source: BMC Genomics. 2019 Apr 8;20:276. doi: 10.1186/s12864-019-5631-3 (PMC6454663; doi:10.1186/s12864-019-5631-3)
Supplement: Supplementary file 6 — Figure S6. A false positive example of RMalign benchmarking in balance-SCOR. 5msf:S (yellow) is superposed on 2iz8:R (cyan) by RMalign. These two RNA structures are very similar (RMscore = 0.837). But they are belonged to two distinct function classes in FSCOR (Phage_coat_protein_binding, MS2_phage_coat_protein_binding_stem-loop). (PDF 236 kb) [file 12864_2019_5631_MOESM6_ESM.pdf]

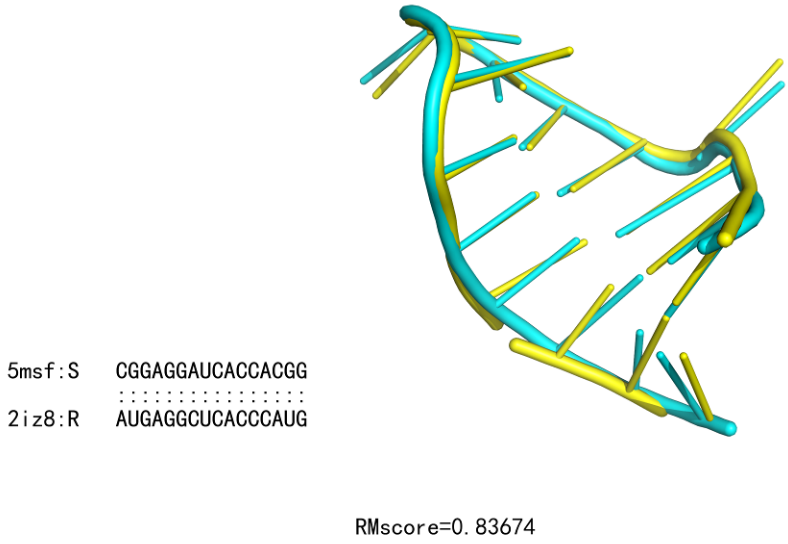

Figure S6. *A false positive example of RMalign benchmarking in balance-SCOR.* 5msf:S (yellow) is superposed on 2iz8:R (cyan) by RMalign. These two RNA structures are very similar (RMscore = 0.837). But they are belonged to two distinct function classes in FSCOR (Phage_coat_protein_binding, MS2_phage_coat_protein_binding_stem-loop).
