# Supplementary figures and images for: RMalign: an RNA structural alignment tool based on a novel scoring function RMscore
Source: BMC Genomics. 2019 Apr 8;20:276. doi: 10.1186/s12864-019-5631-3 (PMC6454663; doi:10.1186/s12864-019-5631-3)

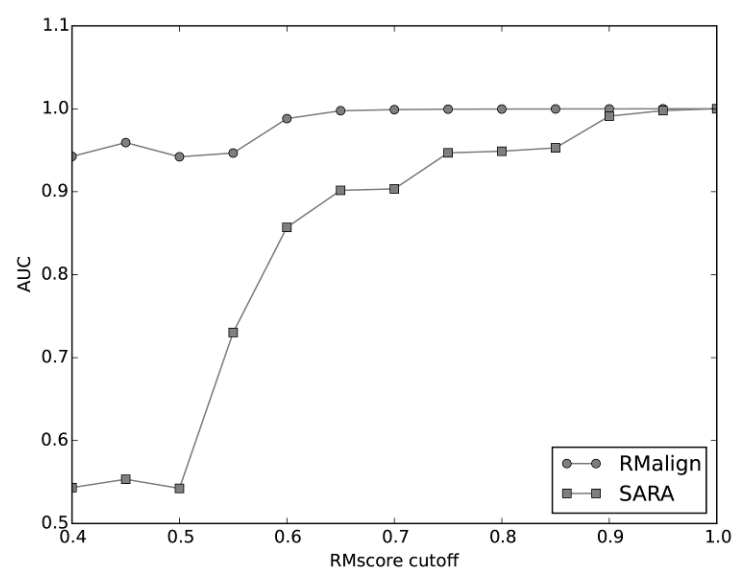

**Figure S7.** Benchmarking of *RMAalign* and *SARA* in *balance-x-FSCOR*.

Supplement: Supplementary file 7 — Figure S7. Benchmarking of RMalign and SARA in balance-x-FSCOR. (PDF 143 kb) [file 12864_2019_5631_MOESM7_ESM.pdf]
